# Eye Donation: Awareness, Knowledge, Willingness, and Barriers among Paramedical and Allied Health Science Students at a Tertiary Care Teaching Hospital in South India

**DOI:** 10.1155/2022/5206043

**Published:** 2022-02-23

**Authors:** Aimanfatima Kacheri, Rekha Mudhol, Sanjeev Chougule, Rhema Reny, Sagarika Kamath, Rajesh Kamath

**Affiliations:** ^1^KLE University, Belagavi, Karnataka, India; ^2^Prasad Medical College, BLDE Deeemed to be University, Vijaypur, India; ^3^Department of Hospital Administration (MBA), J. N. Medical College, KLE University, Belagavi, Karnataka, India; ^4^Prasanna School of Public Health, Manipal Academy of Higher Education, Manipal, India; ^5^Manipal Institute of Management, Manipal Academy of Higher Education, Manipal, India

## Abstract

**Background:**

Visual impairments have physical, emotional, social, and economical consequences and are a crucial element influencing one's quality of life. A total of 1.285 million people are estimated to be visually impaired worldwide of which 39 million are categorised as blind. These figures are startling, given that 80 percent of known vision impairments are either treatable or preventable. Corneal transplants appear to be our best hope for resolving this problem; however, a global shortage of available donors continues to dampen efforts addressing this issue.

**Methods:**

This two-year cross-sectional study employed a convenience sampling technique and a standardised questionnaire to survey 150 paramedical and allied health science students at a tertiary care teaching hospital and assessed the awareness, knowledge, willingness and barriers regarding eye donation.

**Results:**

The study revealed a 93.3% awareness rate of the donation procedure, of which 46% attributed their awareness to media sources. However, other aspects assessed had much lower awareness rates; when the eyes are donated (53.3%), optimal time period for retrieval of tissue/organ (54%), ideal part transplanted (54%), age limit not restricting donation (67%), donation by donors using spectacles (48%), confidentiality of the donor and recipient (54%), hospital having the facility of an eye bank (63%). 49 percent of the respondents were willing to pledge themselves as eye donors, and a majority of the unwilling respondents reported that familial opposition was the reason for their hesitation.

**Conclusion:**

Knowledge levels appear to be below expectations, and more effort is required to ensure that knowledge is imparted to our healthcare practitioners, who will then transfer this knowledge to the population, resulting in an increase in donation rates.

## 1. Introduction

Eye donation involves the recovery, preparation, and delivery of donated eyes for corneal transplants and research. The first successful corneal transplant was performed in 1905, and the first eye bank was founded in 1944. Organ donation found its breakthrough when Doctor Joseph Murray performed a kidney transplant procedure in which Ronald Lee Herrick donated a kidney to his identical twin brother in 1954, making it the first-ever organ donation. He later went on to win the Nobel Prize in Physiology or Medicine in 1990. Organ donation in India is governed by the Transplantation of Human Organs and Tissues Act, which provides the legal structure guiding organ donation for both the deceased and the living. According to the World Health Organization, the percentage of organ donation in India is 0.01%.

Vision is the ability to perceive the surrounding world using light in the visible spectrum reflected by the objects in the environment and any diminishment of function results in visual impairment or blindness ranging from partial to complete with varying visual acuity.

According to estimates provided by the World Health Organization (WHO), someone goes blind every five seconds, which is extremely alarming when 80% of known visual impairments are either curable or preventable [1]. A total of 1.285 million people are estimated to be visually impaired worldwide of which 39 million are categorised as blind. Developing countries account for 80% of the world's blind population. The yearly global costs of productivity losses related to vision impairment from untreated myopia and presbyopia alone are estimated to be US$ 244 billion and US$ 25.4 billion, respectively, due to vision impairment [2].

A finding of the recent global survey performed on eye banking and corneal transplantation computed that there is only 1 cornea available for every 70 corneal recipients worldwide, which shows that the world is experiencing a drastic mismatch between the demand and supply of donor corneas [3].

Corneal transplantation remains the best available option for visual rehabilitation. Based on the current accessibility of donor eyes and its consumption rates, it is projected that 2,70,000 donor eyes will be needed to complete 1,00,000 corneal transplants per year in India, a fourfold increase over current donor eye availability. To overcome this scarcity of eye donors, a 3-tier community system has been proposed for India, and these are eye donation centres, eye banks, and eye bank training centres that are responsible for collecting, processing, and allocating tissue and creating public awareness as well as training and skills enhancement of eye banking personnel. The EDC is responsible for public and professional awareness of eye banks. It coordinates with donor families and healthcare institutes to stimulate eye donation to harvest corneal tissues. It also collects blood for serology and promotes safe practices in eye transplants [4].

There have been several studies carried out in the past regarding eye donation awareness among the general population. This study focuses on paramedical and allied health science students at a tertiary care teaching hospital who were chosen as they represent our country's young and well-trained cadre, who have full access to newspapers, digital media, and other sources of literature. As future healthcare practitioners, their awareness of aspects related to eye donation should be superior to that of the general public. As they pursue their medical degrees, they will be a viable source for increasing the number of eye donations among patients through patient counselling [5, 6]. The intention of this study is to measure the knowledge, willingness, and barriers regarding eye donation among paramedical and allied health science students and thereby enhance the awareness and importance of promoting this endeavor.

## 2. Methodology

A sample size of 150 was determined for this cross-sectional study of paramedical and allied health science students at a tertiary care teaching hospital, and a convenience sample strategy was used to select participants and gather data.

  Inclusion criteria: Students belonging to the DMLT, DOT, BPH, MHA, MLTC, MPH, nutrition and dietetics, and perfusion technology health sciences streams and those who are willing to participate in the study.
  Exclusion criteria: All students belonging to health science streams not previously specified and those students that are not willing to participate in the study.

The research took place over a two-year period, from January 2020 to December 2021. Written consent and the demographic details of the respondents were obtained prior to their participation. Questionnaires were distributed to the participants and collected to be analysed over a three-month period. The standardised questionnaire consisted of 17 closed-ended questions and was divided into two sections: the first assessed participants' awareness and knowledge of eye donation, and the second assessed their willingness to donate their eyes as well as any barriers that would prevent them from doing so. MS Excel version 10 was used to compile and analyse the data, and the results were obtained using percentage and descriptive statistics.

## 3. Results

This section describes the demographic characteristics of the respondents.

**Figure 1:**
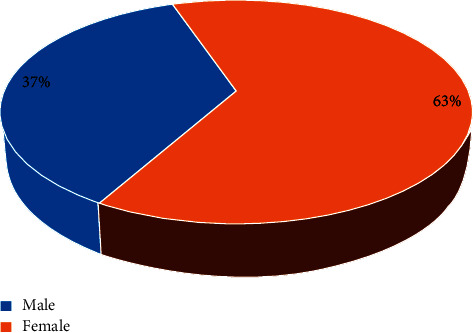
Distribution of students according to their gender.

The male-to-female gender distribution in the respondent's pool was 36.67 percent to 63.33 percent as represented in Table 1 and Figure 1.

**Figure 2:**
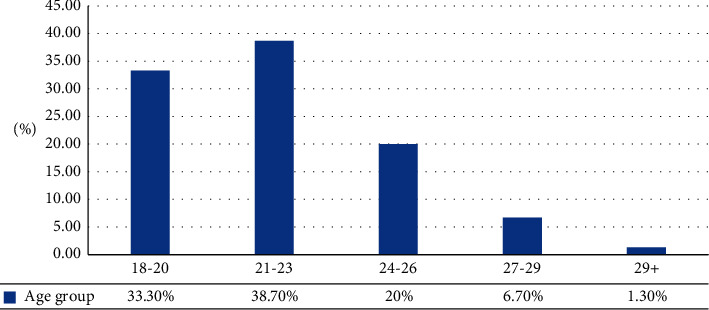
Distribution of students according to their age group.

The age distribution of the participants was as follows: 33.3% (50) of them belong to the age group of 18–20 years, 38.7% (58) of them belong to the age group of 21–23 years, 30 of them belong to the age group of 24–26 years, 10 of the participants belong to the age group of 27–29 years, and only 2 of them are above 29+ year age group as represented in Table 2 and Figure 2.

**Figure 3:**
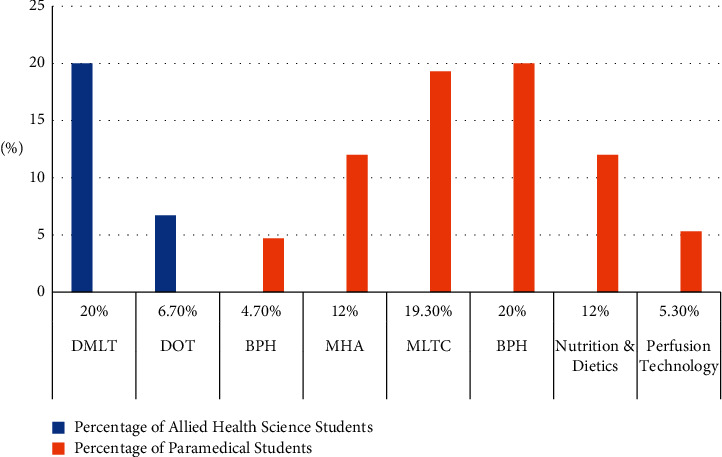
Distribution of students based on their respective courses.

Of 150 students, 110 students belong to Allied health science, 7 (4.3%) students belong to BPH, 18 (12%) students belong to MHA, 29 (19.3%) students belong to MLTC, 30 (20%) students belong to MPH, 18 (12%) students belong to nutrition and dietetics, and 8 (5.3%) students are from perfusion technology. The remaining 40 students belong to the paramedical field, of which 30 (20%) students belong to DMLT and 10 (6.7%) students are from DOT as represented in Table 3 and Figure 3.

The following section describes the findings of the study:

Out of 150 students, 93.3% (140) students are previously aware of the eye donation procedure. Majority of the students 46% (69) responded that mass media is their source of information, 26% (39) said that they got to know about eye donations through lectures, 11.3% (17) of the students gained insight through organ donation campaigns, about 3.3% (5) of students mentioned doctors as their source of information, 10.7% of students identified the hospital/clinic as their source of information about eye donations, and 2.7% (4) were not able to identify a particular source. Eighty students were aware eye donation is carried out only after death, and majority, 54% (81), were well aware of the optimal time for retrieval of eyes after death. 63% (94) of students were aware that their hospital has an eye bank, and 18% (27) knew of a person who has donated their eyes. Thirteen (9%) students responded that the whole eye can be transplanted, 4 (3%) said the lens is transplanted, and 85 (56%) were aware that the cornea is transplanted, while 42 (32%) were not aware of which part of the eye is transplanted during eye donation. 66.67% (100) of the students were unaware there is no age limit restricting the donation of eyes, and 52% (78) were unaware that people using spectacles can donate their eyes. 81% (122) were aware that eye donation requires prior consent, and 71% (106) were well aware that the donor's family will not be charged for the donation. 44% (66) were aware that eye donation can't cure all types of blindness. 54% (81) of the students were aware that the name of donors and recipients remain unknown, whereas the 46% (69) who responded “No” were unaware of the confidentiality maintained. Regarding awareness on scope of benefits from one donation 36% (54) responded that one person is benefitted, 11.3% (17) responded that more than two people are benefitted and majority 52.7% (79) responded that exactly two people are benefitted. These data are represented in Table 4.

Of 150 students, 73 (49%) were willing to pledge their eyes and 77 (51%) were unwilling to pledge their eyes. 14% (11) stated “lack of awareness,” 63% (48) stated “objection by family,” 22% (17) stated “unacceptable idea of the removal of eyes,” and 1% (1) stated that they will be born blind in the subsequent birth as reasons preventing them from registering as donors. A majority of them (37.7% (29)) attributed their response to family opposition, 13% (10) attributed it to perceived effects in the future, 27.3% (21) found the procedure complicated, 14.3% (11) feared that organs would be missed, 1.3% (1) believed the distortion of physical appearance was a barrier for eye donations, and 5% (5) found that it to be a clear violation of human rights, as represented in Table 5.

## 4. Discussion

According to the findings of this study, 93.3 percent (140) of participants were aware of eye donations, which is still shy of the required 100 percent optimal result considering the healthcare background of the participants. A similar study conducted by Sushma et al. [1] among medical and paramedical students in tertiary care hospital revealed a 99.2 percent awareness rate, which is considered to be much closer to a desired result. It is necessary to make an effort to uncover knowledge and awareness gaps in students, particularly those from healthcare institutes, and to shift education from a syllabus-oriented model to a wholistic intellectual approach.

When asked about the sources of their awareness regarding eye donation, majority of the participants (46 percent) stated that the media is their primary source of information. The accelerated advancements in technology and increased access to the Internet have made it possible for the younger generation to easily gather knowledge. This demonstrates the significant impact that media has on the youth. Similarly, a study carried out by Nekar et al. [7] among dental students of KIST Medical College, Nepal revealed that 69.1 percent of the participants identified media to be their primary source of information (44.8% from television and 24.3% from newspapers). [7] Access to information is no longer an issue, but ensuring that the proper information reaches the intended audience has become a major hurdle due to the introduction of a plethora of disruptions. The intended message is lost either in translation or in the sheer vastness or amount of material disseminated. These aspects should be kept in mind, particularly while developing educational resources.

A study conducted by Williams and Muir [8] to assess the awareness and perception of eye donation among medical staff brought to light that only 0.5% of study participants knew of a person who had donated their eyes. The data from this study showed a slight improvement in numbers, with 18% (27) of the students having first-hand knowledge of someone who had donated their eye. Personal experiences create personal beliefs, and public opinion cannot be impacted for the better until we emphasise on known examples in our communities. Examples of prominent members of society who have pledged or donated their eyes can be used to inspire others to do the same.

56% (85) of the participants knew that the cornea is transplanted during the donation of eyes, which seems to be a slight improvement when compared to a study carried out by Lal et al. [9] on “Awareness of eye donation among college students of Hubli city” in which only 33% of the participants were aware that the cornea is harvested during eye donation. 44% (66) of the participants were aware that eye donation is not the cure for all types of blindness, and 52% (79) of the students knew that the scope of benefits from one donation can extend to two unsighted persons. Understanding the full extent of benefits is critical since it will serve as a powerful drive for donations. Well over 50% of the families of the deceased can be motivated to donate their eyes by a well-trained eye donation counsellor [10], and thus, it is critical that due diligence be given to the development of such skilled personnel.

53 percent (80) of the students were aware that eyes can be donated only after death, and it is much higher when compared to a study assessing awareness and willingness of eye donation among paramedical workers carried out by Rangu et al. [11], which revealed that only 38% were aware of the procedure being undertaken after death. 54% (81) of the students were aware of the optimal time within which the donor eye will have to be retrieved; that is, 6 hrs and 67% (100) of students were aware that age limit was not a restriction for eye donation. It was also observed that only 63% of students were aware that their hospital has an eye bank and 54% (81) of the participants knew that the identity of the donor and recipient would be kept confidential. Similarly, in the study conducted by Rangu et al. [11], only 40% participants knew that the donor and recipient names would remain confidential. These figures indicate that we are barely halfway of the optimal goal of achieving 100% awareness among the sampled healthcare professionals. This can be accomplished by re-emphasising the importance of interdepartmental communication and inclusion, which is critical in a diverse healthcare environment that has developed departmental silos over time.

A higher percentage of students (81%) were aware that prior consent was required and that the family would not be charged for the donation (71%). These seemingly higher awareness rates could be attributed to the fact that the participants belonged to the medical field and were routinely involved in obtaining consent prior to any procedures.

In this study, the most common reason for not pledging eyes was objection from family, which accounted for 63 percent of the respondents. This differs from similar studies carried out by Basnet et al. [12] & Williams and Muir [8] in which students attributed their refusal majorly to the lack of information and awareness, respectively. Although 49% of the respondents responded that they would be willing to pledge themselves as an eye donor, the question still remains as to how many donations would follow when the time came, given the societal and familial norms that act as barriers. India, an inherently traditional society, deems the familial unit to be a sacred institution. Many pledges may not be translated into transplants if they are left to the discretion of relatives. Fundamental religious beliefs of reincarnation dominate the majority. This reinforces the belief system in individuals who conform to the collectivism found in eastern societies, which social scientists describe as influencing decisions affecting most aspects of life. [13].

## 5. Conclusion

Allied health science and paramedical students are the future of our healthcare system, since they will be the pillars that sustain it, and their degree of knowledge and awareness is crucial in promoting eye donation. When these healthcare professionals are well informed about the implications and benefits of encouraging eye donations, they will in turn counsel family members to donate their eyes. Educated families will then transfer this responsibility by shaping public views, thereby building a supportive community. However, failure to transfer such knowledge can have disastrous consequences, as many potential donors may be lost. Current corneal donation rates are insufficient to meet India's transfer needs, and each missed opportunity adds to this growing quandary.

This study is extremely important as it illustrates the lack of awareness among our young healthcare professionals. Much effort is required in imparting knowledge to bring about effective change. Media is indeed a powerful tool in ensuring that knowledge is communicated in a way that has a lasting impact on the recipients and should be used in raising awareness. Similar studies are warranted to analyse the widespread lack of awareness so that it can be effectively addressed.

We take pride in family ties and social norms instilled in us by our cultural heritage, but they are inevitably a major impediment to an effective increase in donation rates. The perception of one's experiences shapes a person's belief system, and within a community, similar shared experiences tend to establish a shared belief or value system. We must endeavor to not only break stigmas but create values that will inspire benevolence and humanitarianism. Positive reinforcement such as government or private initiatives will drive behaviors that will help to foster an organ donation culture. Policies mandating a structured dual counselling process, which would include an initial request made by the attending medical personnel, followed by a referral provided by an eye bank personnel at the sources for eye donation, would be extremely beneficial in converting pledges to actual donations, as it would ensure all healthcare facilities, emergency departments, morgues, and funeral homes to actively engage in the donation [10].

## Figures and Tables

**Table 1:** Student distribution according to their gender.

Gender	Number	Percentage
Male	55	36.67
Female	95	63.33
Grand total	150	100

**Table 2:** Distribution of students according to their age group.

Age	No. of participants	Percentage
18–20	50	33.3%
21–23	58	38.7%
24–26	30	20%
27–29	10	6.7%
29+	2	1.3%
Grand total	150	100.0

**Table 3:** Distribution of students based on their respective courses.

Courses	Allied health science students	% of allied health science students	Paramedical students	% of paramedical students	Grand total
DMLT	—	—	30	20	30
DOT	—	—	10	6.7	10
BPH	7	4.7%	—	—	7
MHA	18	12%	—	—	18
MLTC	29	19.3%	—	—	29
MPH	30	20%	—	—	30
Nutrition and dietetics	18	12%	—	—	18
Perfusion technology	8	5.3%	—	—	8
Grand total	110	73%	40	27%	150

**Table 4:** Participants' awareness and knowledge of eye donation.

Sl No	Topic	Response	No.	%
1	Awareness of eye donation	Yes	140	93.3
		No	10	6.6

2	Sources of awareness	Mass media	69	46
		Lectures	39	26
		Organ donation campaigns	17	11.3
		Doctor	5	3.3
		Hospital/clinic	16	10.7
		Don't know	4	2.7

3	Awareness of eyes being donated after death	Aware	80	53.3
		Unaware	70	46.7

4	Age limit for eye donation	Yes	50	33.3
		No	100	66.6

5	Optimal time for retrieval of eyes after death	Within 6 hrs.	81	54
		As soon as possible	25	16.7
		Within 24 hrs./week	5	3.3
		Don't know	39	26

6	Knowledge about ideal part transplanted	Entire eye	13	9
		Lens	4	3
		Cornea	85	56
		Don't know	48	32

7	Awareness of the hospital having an eye bank.	Yes	94	63
		No	56	37

8	Personal knowledge of an eye donor	Yes	27	18
		No	123	82

9	Awareness regarding donation by donors using spectacles	Yes	72	48
		No	78	52

10	Prior consent required for eye donation	Yes	122	81
		No	28	19

11	Awareness on charges associated with eye donation	Yes	44	29
		No	106	71

12	Awareness on eye donation not being the cure for all types of blindness	Yes	66	44
		No	84	56

13	Awareness on confidentiality of the donor and recipient	Yes	81	54
		No	69	46

14	Awareness on scope of benefits from one donation	1 unsighted person benefitted	54	36
		More than 2 unsighted persons benefitted	17	11.3
		Two unsighted persons benefitted	79	52.7

**Table 5:** Willingness and barriers related to eye donation.

1	Willingness of the participants to donate/pledge their eyes	Willing to pledge	73	49%
		Unwilling to pledge	77	51%

2	Reasons for not pledging/donation of eyes	Lack of awareness	11	14%
		Objection by family	48	63%
		Unacceptable idea of the removal of eyes	17	22%
		Will be born unsighted in the subsequent birth	1	1%

3	Barriers for eye donation	Family opposition	29	38%
		Affects the future	10	13%
		Complicated nature of procedure	21	27%
		Misuse of organs	11	14%
		Affected physical appearance	1	1%
		Insults human rights and dignity	5	6%

## Data Availability

Data are available from authors on request.
